# A portable optical pulsatile flowmetry demonstrates strong clinical relevance for diabetic foot perfusion assessment

**DOI:** 10.1063/5.0182670

**Published:** 2024-02-21

**Authors:** Renzhe Bi, Ruochong Zhang, Lingyan Meng, Yao Du, Julie Low, Yi Qi, Poongkulali Rajarahm, Alexis Yuen Fun Lai, Victoria Shi Ying Tan, Pei Ho, Malini Olivo

**Affiliations:** 1 A^*^STAR Skin Research Labs (A^*^SRL), Agency for Science, Technology and Research (A^*^STAR), 31 Biopolis Way, Nanos, Singapore 138669, Republic of Singapore; 2 National University Health System (NUHS), 1E Kent Ridge Rd., Singapore 119228, Republic of Singapore; 3 National University of Singapore, Yong Loo Lin School of Medicine, 10 Medical Dr, Singapore 117597

## Abstract

We present a robust, cost-effective (<2000 USD), and portable optical diffuse speckle pulsatile flowmetry (DSPF) device with a flexible handheld probe for deep tissue blood flow measurement in the human foot as well as a first-in-man observational clinical study using the proposed optical device for tissue ischemia assessment and peripheral artery disease (PAD) diagnosis. Blood flow in tissue is inherently pulsatile in nature. However, most conventional methods cannot measure deep tissue-level pulsatile blood flow noninvasively. The proposed optical device can measure tissue-level pulsatile blood flow ∼6 mm underneath the skin surface. A new quantitative tissue perfusion index (TPI_DSPF_) based on frequency domain analysis of the pulsatile blood flow waveform is defined to assess tissue ischemia status. Through a clinical study involving 66 subjects, including healthy individuals and diabetes patients with and without PAD, TPI_DSPF_ demonstrated strong correlations of 0.720 with transcutaneous tissue partial oxygen pressure (TcPO2) and 0.652 with toe–brachial index (TBI). Moreover, among the three methods, TPI_DSPF_ demonstrated the highest area under the curve for PAD diagnosis among diabetes patients, with a notable value of 0.941. The promising clinical results suggest that the proposed optical method has the potential to be an effective clinical tool for identifying PAD among the diabetic cohort.

## I. INTRODUCTION

Diabetes mellitus (DM) is a chronic disease that is projected to affect 578 × 10^6^ people worldwide by 2030.^1^ Peripheral artery disease (PAD) is highly associated with type 2 diabetes, and up to 50% of patients with type 2 diabetes will likely develop PAD through their lifetime.^2^ PAD can lead to claudication, tissue ischemia, foot ulcers, and significantly decreased life quality.^3^ Diabetic foot ulcer (DFU) is a common complication for DM patients with PAD.^4,5^ PAD also frequently presents in patients with DFU and was reported to be around 50% in prior study.^6^ When both PAD and DFU occur, wound healing becomes much more difficult. Risk of major limb amputation for patients with both PAD and DFU is also substantially higher than when only one condition is present.^7^ Mortality risk for DM patients with major amputation can be as high as 52%–60%.^8^ Apart from macro-vascular disease like PAD, DM patients have been found to have increased thickness of capillary basement membrane, reduced arteriolar luminal area, and reduced capillary density.^9^ Such changes contribute to delayed would healing. An easy-to-perform noninvasive screening of perfusion impairment is important to diagnose PAD early in diabetic individuals before clinical symptoms manifest. This will enable proper risk stratification among all the asymptomatic DM patients and guide clinical management decisions for DFU patients.

Current PAD diagnosis and assessment methods for DM patients include palpation of pulses,^10,11^ toe–brachial index (TBI) measurements,^10,12^ and transcutaneous tissue partial oxygen pressure (TcPO2) measurements.^10,13,14^ Absence of pulses in dorsalis pedis or posterior tibial arteries is a strong indicator for PAD.^11^ However, palpation of pulses in the foot requires experienced clinicians and yields only subjective (absence or presence) result. In the latest Wound, Ischemia, and foot Infection (WIfI) Classification System for lower extremity wounds, TBI is a preferred measurement to determine ischemia grade for foot wound patients.^15^ TBI also demonstrates a good sensitivity in detecting PAD among DM patients,^4,6^ but it has a large error range.^12,16^ Its value of diagnosing PAD is impaired for patients with missing toes, ulcer over the toes, or with ulcer over the hind-foot region. In those situations, TcPO2 is suggested as an alternative measurement for tissue ischemia assessment.^15^ TcPO2 measures partial pressure of tissue oxygen and is a predictor for foot ulcer healing potential. A TcPO2 > 40 mmHg strongly correlates with increased wound healing.^17,18^ However, resting TcPO2 is insufficiently sensitive for early stage PAD^19^ detection, and it requires expensive equipment, well trained operators, and long acquisition time (∼40 min) to perform. Its readings may be affected by ambient temperature, body position, tissue edema, patients' caffeine intake, and smoking habits.^20^ TcPO2 with high-cost instrumentation and a sophisticated process for acquiring the reading is only available in some specialized tertiary healthcare institutions and not in primary health care where the greatest clinical needs exists. Therefore, a new noninvasive and easy-to-operate screening device is highly needed.

Pulsatile blood flow to tissue is critical because it brings necessary oxygen and nutrition. Pulsatile flow is known to result in more homogenous tissue perfusion.^21^ Peripheral perfusion index (PPI), which is defined as the ratio between the pulsatile and non-pulsatile components from the pulse oximeter signals, has been used for monitoring critically ill patients.^22,23^ However, pulse oximetry measures changes in blood volume and is limited to a few monitoring sites such as fingers, toes, and ears. Whereas doppler ultrasound is employed to assess blood flow in both major and minor blood vessels, it is not suitable for evaluating blood flow at the tissue level.^24^ Laser speckle contrast imaging (LSCI) is a popular technology for blood flow imaging, but it is mainly limited to that of the superficial tissue within 1 mm depth.^25^ Diffuse correlation spectroscopy (DCS) is a well-known technology for deep tissue blood flow measurement.^26^ Although recent development of DCS allowed for *in vivo* pulsatile blood flow measurement at 20–50 Hz rate,^27–30^ the experiments were only demonstrated on an animal model^30^ and on healthy subjects in a laboratory environment.^27–29^ Increasing the sampling rate would decrease the signal to noise ratio (SNR) and affect its accuracy.^27^ In a recent clinical study for PAD research, a DCS measurement rate of 0.13 Hz was used,^31^ which failed to measure the pulsatile blood flow. Moreover, DCS devices require relative expensive components like photomultiplier tube and fast data acquisition card. Therefore, the application of DCS devices in clinical settings for pulsatile blood flow measurement is challenging.

Recently, a noninvasive optical method named diffuse speckle pulsatile flowmetry (DSPF) has been developed to measure the blood flow in deep tissue (1–15 mm) at very high measurement rate (> 300 Hz).^32^ The use of a multi-mode (MM) detection fiber can increase signal throughputs.^32,33^ However, the reported DSPF system required a costly laser and was constructed on an optical table, which cannot be used in clinical settings.^32^ In this paper, we present a first-of-kind portable, robust, and cost-effective DSPF device with a flexible handheld probe, as well as a novel tissue perfusion index (TPI_DSPF_) derived from the frequency domain analysis of DSPF readings for foot ischemia assessment and PAD diagnosis.

To validate the tissue ischemia assessment capability of the device and TPI_DSPF_ against the well-established methods, TcPO2 and TBI, a first-in-man observational clinical study was designed and conducted at National University Hospital of Singapore to perform these three measurements on three cohorts including (1) healthy subjects, (2) DM patients without PAD, and (3) DM patients with PAD. This study aims to validate the application of the proposed DSPF device as a diagnostic tool for PAD in DM patients by analyzing the correlation between TPI_DSPF_ and the standardized perfusion investigation tools. Furthermore, a secondary aim is to compare the capability of the TPI_DSPF_ measurement in detecting the presence of PAD, with that of TPI and TcPO2.

## II. RESULTS

We recruited 66 subjects to be measured by TBI equipment, our portable DSPF device, and TcPO2 equipment. There were 15 healthy subjects (15/15 completed all three measurements), 18 DM patients without PAD (18/18 completed all three measurements), and 33 DM patients with PAD (31/33 completed all three measurements; 2/33 completed only TPI_DSPF_ and TcPO2 because they were missing a toe).

**FIG. 1.**
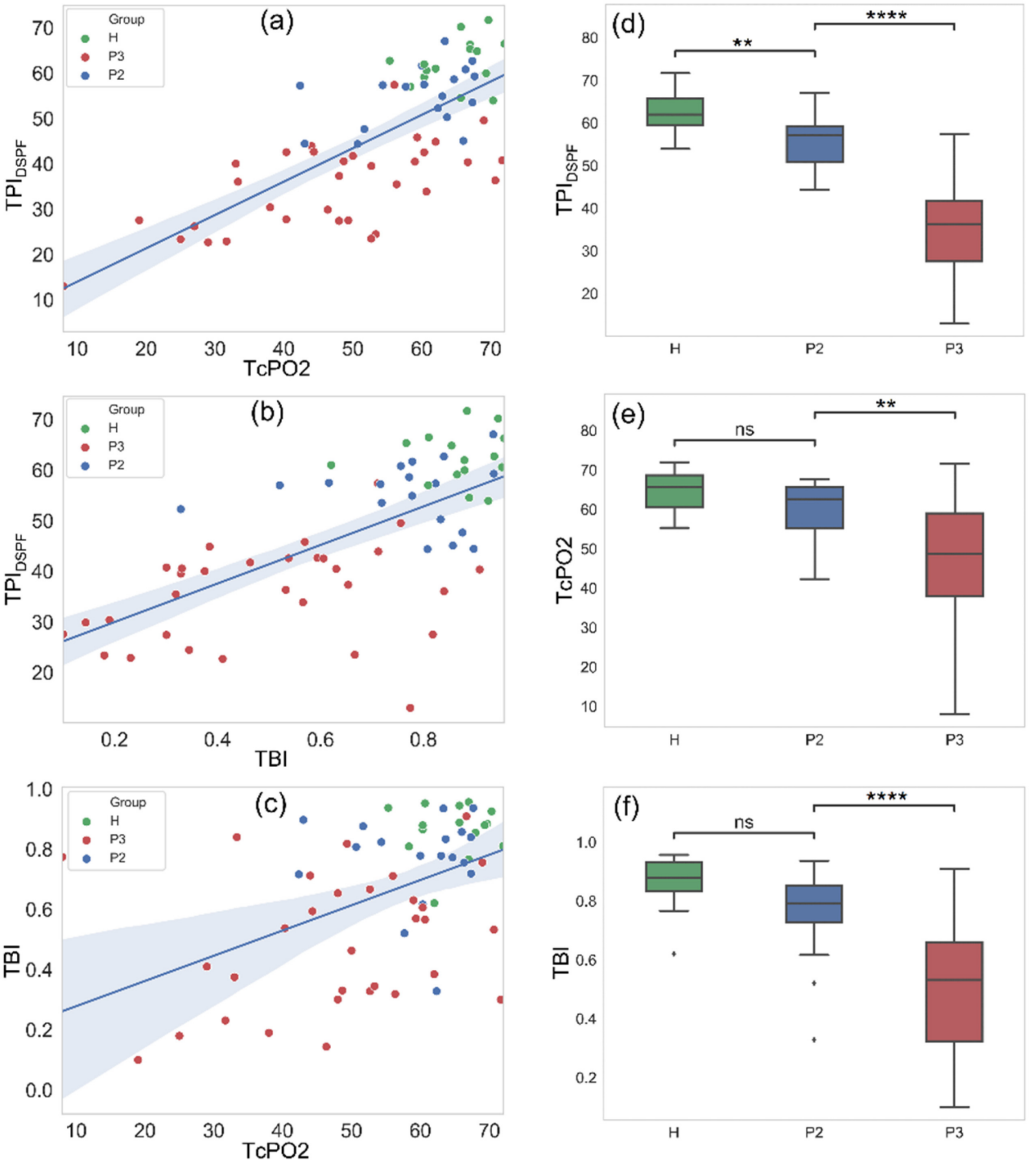
Correlation between (a) TcPO2 and TPI_DSPF_, (b) TBI and TPI_DSPF_, and (c) TcPO2 and TBI. H (green) represents healthy subjects, P2 (blue) represents DM patients without PAD, and P3 (red) represents DM patients with PAD. Pearson's correlation coefficients are 0.720, 0.652, and 0.474 for (a), (b), and (c), respectively. Boxplots of the three groups (H, P2, and P3) under the measurement of (d) TPI_DSPF_, (e) TcPO2, and (f) TBI.

From Figs. 1(a) and 1(b), a strong correlation is observed between TPI_DSPF_ and TcPO2 (Pearson's r = 0.720) for all 66 subjects, and between TPI_DSPF_ and TBI (Pearson's r = 0.652) in the 64 subjects. This result validates TPI_DSPF_'s capability for tissue ischemia assessment over the full physiological range. However, only moderate correlation is observed between TBI and TcPO2 (Pearson's r = 0.474) in Fig. 1(c).

Boxplots of the three groups: healthy subjects, DM patients with and without PAD, are shown in Figs. 1(d)–1(f). The stars above the boxplots indicate the level of statistical significance. All three perfusion assessment methods demonstrated a statistically significant difference in the readings between DM patients with PAD vs the other two groups. TBI and TcPO2 readings did not show any statistically significant difference between healthy subjects and DM without PAD. Only TPI_DSPF_ readings demonstrated a significant difference between healthy subjects and DM without PAD. These results may reveal that the degradation of tissue pulsation due to blood flow is an early indicator of vascular disorder for DM patients. Conversely, the changes in tissue oxygen pressure and toe pressure may only be observed at a later stage.

Compared with TcPO2, TPI_DSPF_ and TBI demonstrate higher statistical significance in differentiation between DM patients with and without PAD, which means TPI_DSPF_ and TBI are more sensitive to PAD than TcPO2 is. Although TBI can differentiate DM patients with and without PAD with high statistical significance, it fails to tell the difference between the first two groups. From our results, there is no significant difference in TBI between healthy subjects and DM patients without PAD.

The measurement results of the three groups, using three different perfusion assessment methods and the demographic information of the subjects are summarized in Table I.

**TABLE I.** TPI_DSPF_, TcPO2, and TBI measurement results (mean, standard deviation, and sample size) of the three groups.

	Healthy control	DM patients without PAD	DM patients with PAD
TPI_DSPF_	61.3 ± 4.2, n = 15	53.5 ± 6.9, n = 18	35.2 ± 10.3, n = 33
TcPO2	67.2 ± 4.6, n = 15	59.6 ± 8.6, n = 18	47.4 ± 15.2, n = 33
TBI	0.86 ± 0.08, n = 15	0.77 ± 0.15, n = 18	0.49 ± 0.22, n = 31
Age	35.6 ± 6.9	59.8 ± 11.5	61.6 ± 10.7
Female/male	9 / 6	8 / 10	4 / 29
Years of DM	NA	19 ± 11.2	20.5 ± 12.5

**FIG. 2.**
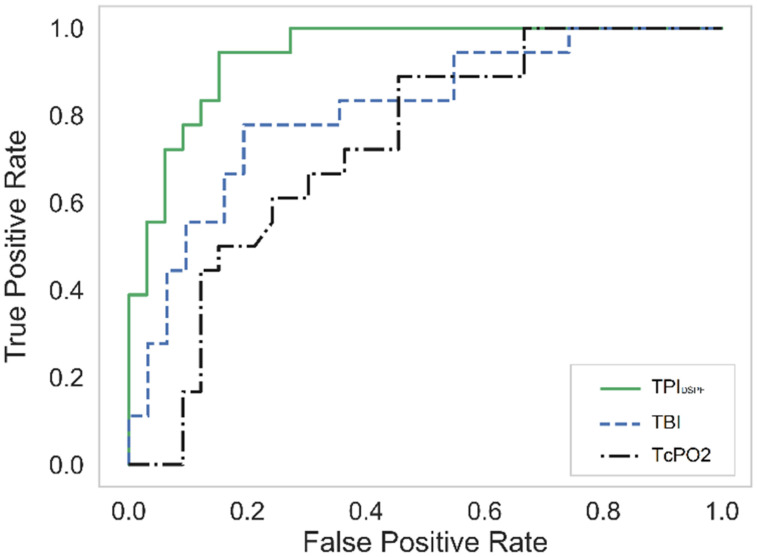
ROC curves of PAD diagnosis among DM patients. The AUC values of TPI_DSPF_, TBI, and TcpO2 are 0.941, 0.812, and 0.730, respectively.

To compare the overall accuracy of the PAD diagnosis among DM patients, the receiver-operating characteristic (ROC) curves of the three methods are shown in Fig. 2. Only the data of DM without PAD (18 subjects) and DM with PAD (33 subjects for TPI_DSPF_ and TcPO2, 31 subjects for TBI) are used for the ROC curves. The AUC values of TPI_DSPF_, TBI, and TcPO2 are 0.941, 0.812, and 0.730, respectively. Under the same condition, TPI_DSPF_ demonstrated the best performance for PAD diagnosis of DM patients among the three methods. The sensitivity and specificity of TPI_DSPF_ in PAD diagnosis of diabetic patients are 94.4% and 84.8%, respectively.

## III. DISCUSSION

Several of our findings in Sec. II are in line with existing literature. Our results indicate that TPI_DSPF_ and TBI are more sensitive to PAD than TcPO2 is. A previous study also confirmed that TBI has a higher accuracy in PAD diagnosis than TcPO2.^19^ Our finding of no significant difference in TBI between healthy subjects and DM patients without PAD is in line with a previous study^35^ showing no significant difference in TBI between the healthy control and the DM patients without any artery disease (Fig. 2 of Ref. 35). Our TcPO2 reading values correspond well with those from previous studies on healthy subjects^36^ and diabetic patients with healing wounds.^18^ Our TBI readings are in the same range as the published data in Ref. 12. The consistency of our clinical measurements in comparison to that of the existing literature verifies the accuracy of our study.

To observe the correlation between the proposed ischemia indicator TPI_DSPF_ and the established clinical methods (TcPO2, TBI), we recruited a spectrum of subjects, including healthy subjects, DM patients without PAD, and those with PAD. TPI_DSPF_ shows a higher correlation with TcPO2 than TBI over all three groups of subjects. This is likely because the measurement locations (three sites over the foot dorsum) for TPI_DSPF_ and TcPO2 were the same, whereas TBI was measured at the big toe, which is a more distal peripheral tissue. Even within the foot, different regions may have different tissue perfusion status, as explained by the angiosome concept,^37^ and this could explain the deviation in correlation between the measurement modalities. Interestingly, although TPI_DSPF_ correlates more with TcPO2, both TPI_DSPF_ and TBI are more sensitive to PAD than TcPO2. This observation may indicate that the tissue pulsation and blood pressure in the toe arterials are early indicators for PAD, while tissue oxygen level may only be affected at a later stage.

A larger number of DM patients with PAD was designed and recruited as this group comprises of patients with different severity of ischemia (TPI ranging from 0.10 to 0.91). The study team expected the perfusion status variation in the other two groups to be relatively less.

This study has a couple of limitations. Due to limited resources, we did not perform longitudinal measurements for the subjects. Furthermore, in the group DM with PAD where a foot ulcer was present, we could not perform longitudinal follow-up to evaluate the wound healing predictive value of TPI_DSPF_. This will be studied in our future work. In some cases, motion artifacts did affect the blood flow measurements. This presented challenges for some subjects who had involuntary foot movements. Additionally, the operator needed to steady the probe meticulously during measurement. In the current study, measurement time for each site was short (∼ 30 s) and limited the amount of motion noise for negligible effect over the frequency domain analysis. Nonetheless, we plan to further improve the mechanical stability of the probe in the future.

## IV. CONCLUSION

This study reports on a robust, cost-effective (<2000 USD), and portable optical DSPF device with a flexible handheld probe, as well as a new indicator, TPI_DSPF_, for tissue-level blood flow assessment of diabetic foot and PAD screening among DM patients. Through a first-in-man clinical study involving 66 subjects, TPI_DSPF_ demonstrated a strong correlation with the current clinical methods, including TcPO2 (Pearson's r = 0.720) and TBI (Pearson's r = 0.652). Moreover, TPI_DSPF_ achieved the highest area under the curve (AUC) of 0.941 for PAD diagnosis in DM patients among the three methods, demonstrating higher diagnosis accuracy than the other two established clinical methods. To the best of our knowledge, this is the first optical method that has been achievable with such superior clinical performance.

Beyond the advantages mentioned earlier, the proposed DSPF device also has the advantages of low laser power (<5 mW), high usability, and short operation time (∼5 min). We envision this to become a useful clinical tool for assessment of tissue ischemia and risk-stratified PAD in the diabetes population.

## V. METHODS

### A. Diffuse speckle pulsatile flowmetry system

**FIG. 3.**
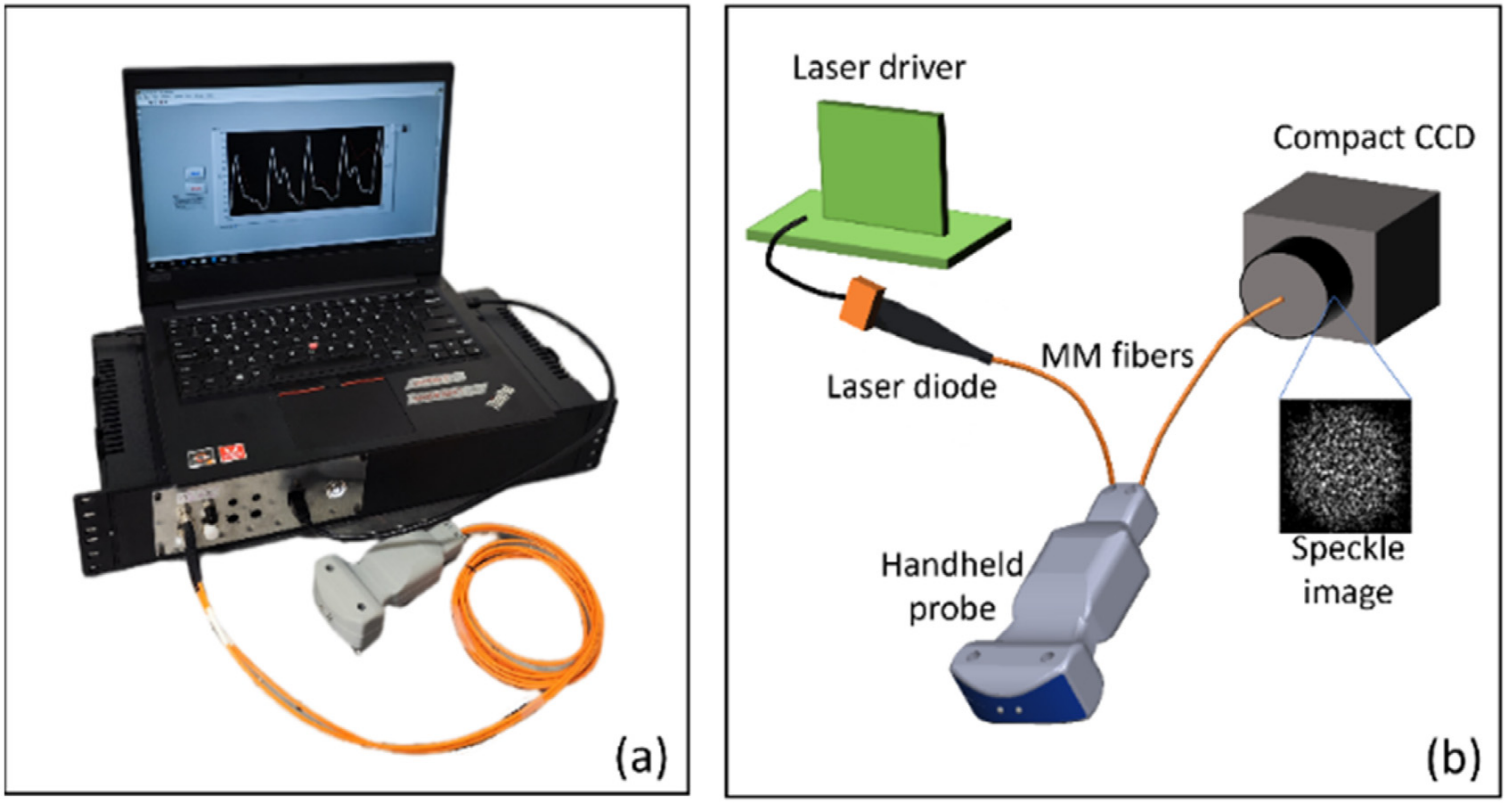
(a) Picture of the portable DSPF system with a fiber-based handheld probe. (b) Simplified schematic of DSPF system.

A portable diffuse speckle pulsatile flowmetry (DSPF) device with a fiber-based handheld probe, as shown in Fig. 3(a) was built. A simplified schematic is shown in Fig. 3(b). The detailed working principle of DSPF can be found in our previous publication^32^ and the supplementary material. In short, an MM detection fiber and a compact CCD are used in DSPF to capture a laser speckle pattern containing multiple speckles. By removing the non-uniform intensity background of the captured speckle pattern from the MM fiber tip, the corrected speckles can be analyzed for blood flow assessment. Comparing with conventional SM detection fiber, where multiple frames of images are required to generate one data point, MM detection fiber only needs one single CCD acquisition to generate one blood flow index (BFI). Therefore, the use of MM detection fiber increases the sampling rate of BFI significantly. Moreover, the high signal throughput of MM detection fiber also allows the use of lower illumination laser power. In the proposed device, the blood flow sampling rate is 330 Hz, and the exposure time for each frame is 2 ms.

In the device shown in Fig. 3(a), a laser diode (785 nm, LP785-SF20, Thorlabs) powered by an OEM driver (EK1101, Thorlabs) served as the illumination light source. The optical power was set to 4.5 mW, which was considered to be a Class 3A laser in Singapore. A compact industrial CCD (Flea3 GigE, Point Grey) was used for signal light detection. Two multi-mode optical fibers with core size of 200 *μ*m were used for light source delivery and signal collection, respectively. The source-detector separation used in this clinical study was 12 mm. A 3D printed handheld probe enclosed the two optical fibers inside, so that the user could hold it by hand comfortably. A cost-effective laptop (AMD Ryzen 5, 8GB RAM, 256GB SSD) controlled the system for data acquisition and display. The hardware cost (<2000 USD) of this portable DSPF device, including the laptop, was very cost-effective.

The proposed device has a compact size (45 × 22 × 10 cm) and weighs light (∼2 kg), which provided high portability for use in community screening centers, primary care, and hospital settings. The advantages of this first-of-its-kind DSPF device include high measurement rate (330 Hz) of blood flow without sacrificing SNR, low laser power (4.5 mW) that is relatively safe for human eyes, flexible and robust MM fiber based handheld probe that is easy to operate, high portability, high cost-effectiveness, and high ease-of-use.

### B. Clinical study protocol

The observational clinical study was conducted in the National University Hospital (NUH), Singapore. All the patient recruitment and measurement were approved by the hospital Domain Specific Review Board (DSRB) (Reference No. 2020/00017). Informed consent was obtained from all participant subjects before the study. This study did not influence the patients' treatment. The inter- and intra-observer reliability were validated during the training phase before the commencement of the study.

**FIG. 4.**
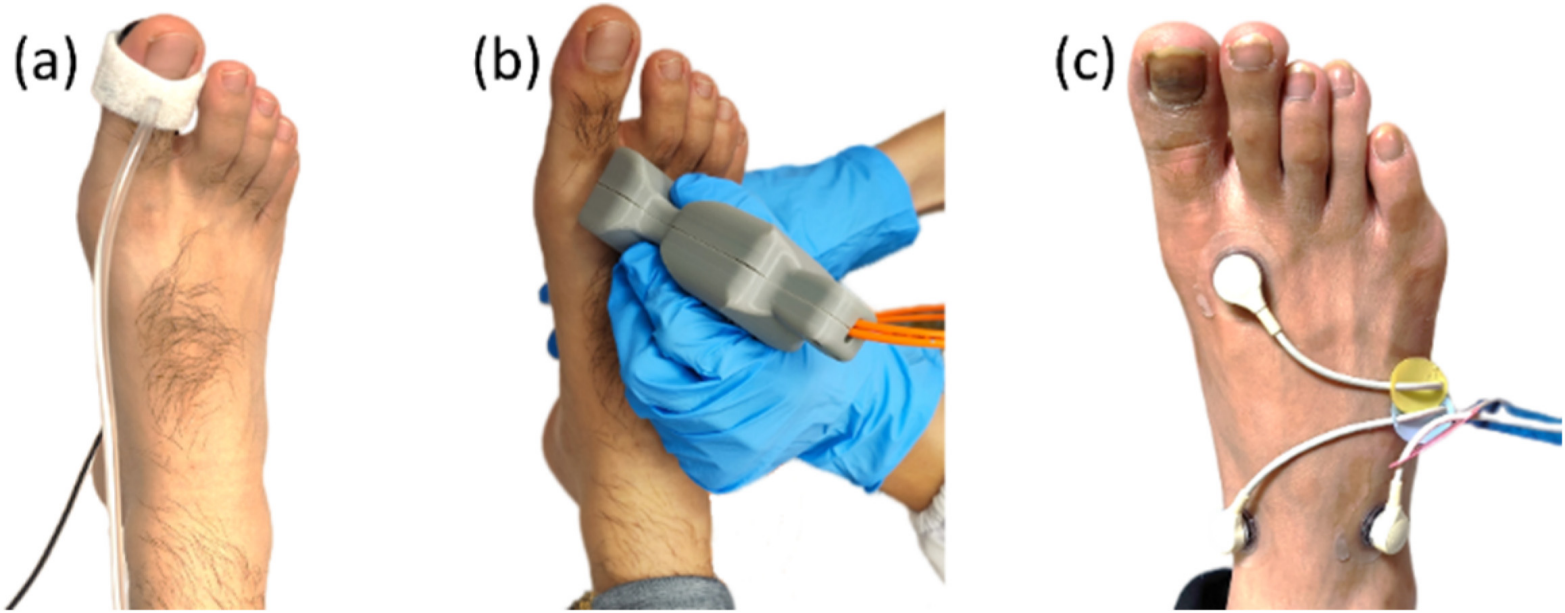
Measurement process for a recruited subject: (a) TBI measurement (only the toe pressure measurement is shown) (b) TPI_DSPF_ measurement, and (c) TcPO2 measurement. Pictures are for illustration purposes only.

Three different groups of participants were recruited: group 1: Healthy volunteers with no comorbidities; group 2: DM patients without history of PAD; and group 3: DM patients with diagnosed PAD. A Patient was considered to have PAD when there was presence of a PAD diagnosis code in the patient electronic record. PAD diagnosis was reached based on either one or more investigations: TPI, ABPI, arterial duplex, CT angiogram, or conventional angiogram. Informed consent was obtained from all participant subjects before the study. The study was conducted with the subject in supine position in a clinical bed with no shoe and sock. The room temperature of the study venue was kept between 21and 23 °C. All participants rested for 10 min before the commencement of measurements. The operator first measured the toe pressure (Hadeco Smartdop® 45) and the brachial pressure (Sphygmotron^TM^) for TBI (toe systolic pressure/arm systolic pressure). Next, the operator DSPF prototype device (built inhouse) probe was used to take measurements of the skin surface at three locations (medial forefoot, medial midfoot, and lateral midfoot) over the dorsal side of the foot. Each location was measured for 30 s. The measurement sites were selected to avoid big blood vessels, and at locations suitable for TcPO2 measurement afterward. The technician was blinded to the results of DSPF. After DSPF blood flow measurement, a four-channel TcPO2 device (Radiometer TCM400) was used to measure TcPO2 level at the same three locations of the DSPF. Since TcPO2 measurement involves warming up of the body tissue to ∼43 °C under the trackpad, which alters the tissue perfusion, we scheduled it to be the last of the three measurements. Readings from the three locations were averaged separately for both the DSPF and TcPO2 measurements to reflect an overall status of the target foot. Illustrative pictures of the three measurements are shown in Fig. 4.

### C. Tissue perfusion index derived from DSPF (TPI_DSPF_)

**FIG. 5.**
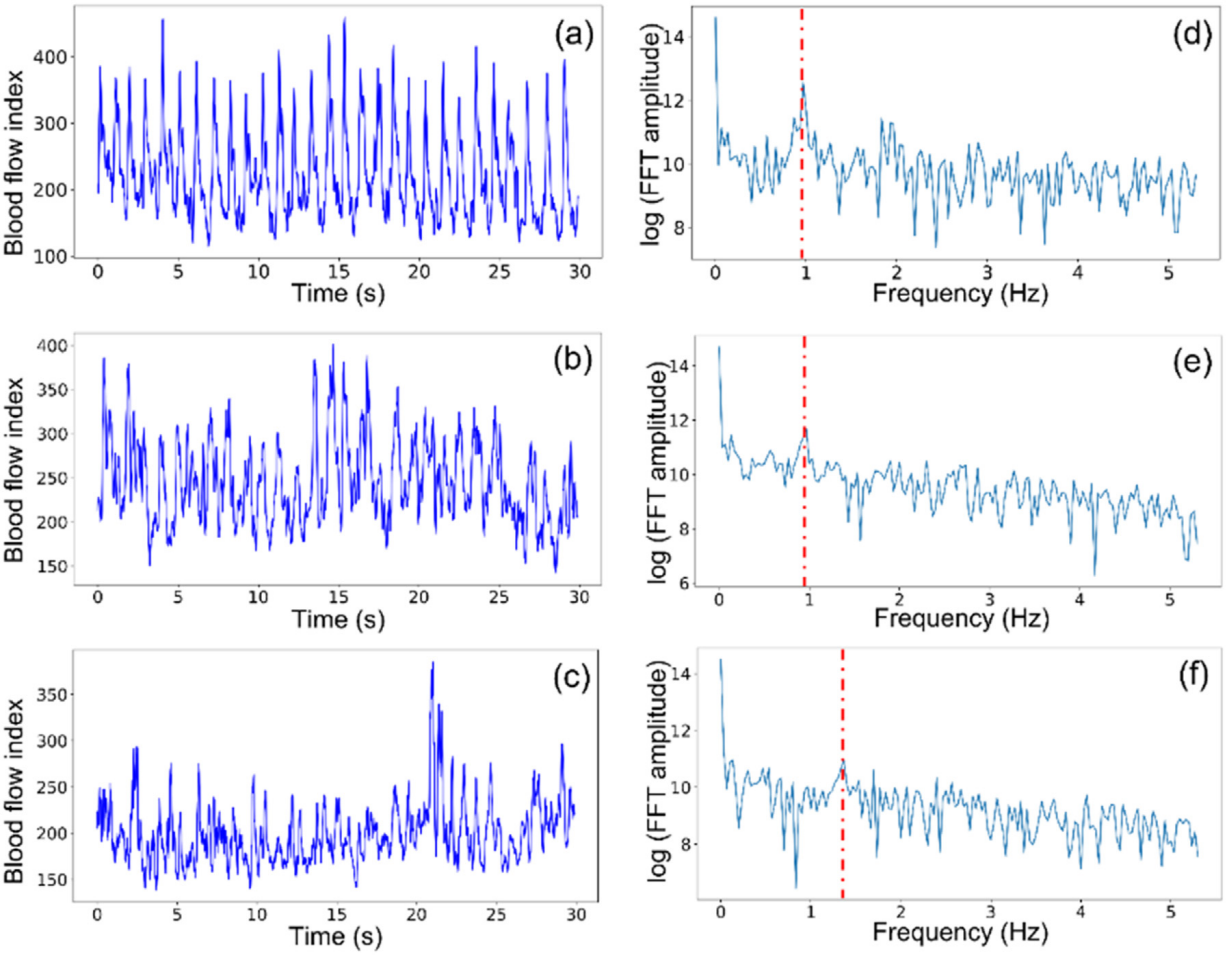
(a)–(c) are pulsatile blood flow waveforms from the foot of a healthy subject, a DM patient without PAD, and a DM patient with PAD and foot ulcer, respectively. (d)–(f) Magnitude plots of the fast Fourier transform of (a)–(c), respectively.

Due to the good sensitivity to deep tissue blood flow of the proposed DSPF device, the pulsatile blood flow waveform from foot tissue can be captured and converted into a new quantitative tissue perfusion index (TPI_DSPF_) based on the frequency domain analysis of the acquired waveform. Pulsatile waveforms of subjects' feet were analyzed using the Fast Fourier Transform (FFT). Examples of blood flow waveforms of a healthy subject, a DM patient without PAD and a DM patient with PAD are shown in Figs. 5(a)–5(c), respectively. Visually, the healthy subject has the most regular pulsatile flow pattern, and the DM patient with PAD has the most noisy and irregular pattern. This can be explained by the narrower and less flexible microvasculature caused by diabetes, leading to reduced blood flow.^34^ To quantify our observations, FFT was performed on the blood flow waveforms. The frequency domain magnitude plots of a healthy subject, a DM patient without PAD, and a DM patient with PAD are shown in Figs. 5(d)–5(f). The Y axis is the log of FFT amplitude. A clear and strong peak at the heartbeat frequency of 0.9 Hz is observed in Fig. 5(d) for the healthy subject. The magnitude at the heartbeat frequency of 1.3 Hz is much weaker in Fig. 5(f) for the DM patient with PAD. The peaks at the heartbeat frequencies of 0.9, 0.9, and 1.3 Hz are indicated by red dash lines in Figs. 5(d)–5(f), respectively.

To quantify the tissue-level pulsatile blood flow, we define the TPI_DSPF_ as follows:

$${\mathrm{TPI}}_{\mathrm{DSPF}}=200\cdot \sqrt{I_{\text{heartrate}}/I_{DC}},$$ (1)

where $I_{\text{heartrate}}$ is the magnitude of the heartbeat frequency component, and $I_{DC}$ is the magnitude of 0 Hz component. We normalize $I_{\text{heartrate}}$ against $I_{DC}$ because the absolute value of BFI is affected by skin color and varying scattering coefficient between individuals. Therefore, the normalization helps to eliminate individual discrepancies caused by differences in skin optical properties. A factor of 200 is multiplied to make the TPI_DSPF_ have a similar value range as that of TcPO2.

## SUPPLEMENTARY MATERIAL

See the supplementary material for movie 1 and the supporting content.

## Data Availability

The data that support the findings of this study are available from the corresponding authors upon reasonable request.
